# Infections Are Not Increased in Scleroderma Compared to Non-Inflammatory Musculoskeletal Disorders Prior to Disease Onset

**DOI:** 10.2174/1874312900701010012

**Published:** 2007-11-08

**Authors:** Janet E Pope, Jodi L Goodwin, Janine M Ouimet, Adriana Krizova, Matthew Laskin

**Affiliations:** Division of Rheumatology, Department of Medicine, The University of Western Ontario, London, Canada

**Keywords:** Scleroderma, etiology, infectious triggers, bacterial infections, viral infections, vaccinations.

## Abstract

The etiology of scleroderma (SSc) is unknown; immunogenic stimuli such as infections and vaccinations could theoretically be risk factors for scleroderma. Our objective was to assess the relationship between viral and bacterial infec-tions, and vaccinations, prior to diagnosis of SSc compared to non-inflammatory controls. Methods: A questionnaire was sent to individuals with SSc (n =83) and controls (n=351) with non-inflammatory musculoskeletal (MSK) disorders (os-teoarthritis, n = 204; tendonitis, n = 58; fibromyalgia, n= 89) from a rheumatology practice. Questions ascertained past in-fections, exposure to infectious agents and vaccination history. Results: The response rate was 78% (SSc) and 56% (MSK controls). The mean age was 56 ± 1.6 (SSc) and 58 ± 0.9 (MSK); 88% (SSc) and 82% (MSK) were female. No association between prior infections and SSc was observed. In fact, controls were more likely than SSc subjects to report any infec-tion within 1-year prior to disease diagnosis (35% vs. 16%, p<0.006), or to have suffered a trauma to affected joints prior to diagnosis (44% vs. 19%, p<0.0002). Within the 1-year prior to disease diagnosis, controls reported slightly more strep-tococcal infections (p<0.2), infections with diarrhea and vomiting (p<0.3), and antibiotic use (p<0.09), although none of these results were statistically significant. Histories of any hepatitis, rubella, any bacterial infection, and having had a pre-vious positive tuberculosis skin test were not significantly different between groups and were actually more often reported by the control subjects. SSc reported slightly more hepatitis B (p<0.08), more rheumatic fever (p<0.8) in past, and herpes zoster (p<0.4), although no differences reached significance. Conclusion: This study does not support that self-report of symptomatic infections are more likely to occur ever (prior to diagnosis) or within 1-year prior to symptom onset of SSc, or that vaccinations in adulthood trigger SSc.

## INTRODUCTION

Systemic sclerosis (SSc) is a connective tissue disorder characterized by excessive deposition of collagen in the skin and multiple internal organs [1]. There is some debate as to whether SSc is primarily an autoimmune disease or whether a positive ANA test is an epiphenomenon of the condition. Thus far, immunosuppressive agents have not been very successful in treating SSc [2]. The etiology of scleroderma remains unknown, though both genetic and environmental factors have been implicated [3]. Reports of familial occurrences of SSc are infrequent, but can occur, and studies investigating an underlying genetic predisposition have used serological techniques to suggest linkages between SSc and HLA-A1;B8;DR3;DR52 haplotypes in certain ethnic populations such as the Choctaw Indians in Oklahoma and Japanese subjects [4]. Furthermore, major histocompatibility complex (MHC) analyses have revealed strong associations of HLA class II alleles with serologically and clinically defined subsets of SSc [1,5].

Early case studies described SSc and acid-fast bacterial infections [6-8]. We could also postulate an altered immune system or unknown co-factor increasing the risk of both tuberculosis (TB) and SSc. Circumstantial evidence for a probable infectious cause (*via* an immune cross-reactivity mechanism where increased activation of a gene regulating the synthesis of a heat shock protein (HSP) in SSc fibroblasts) has been described [9]. Others reported that bacterial HSPs and serum antibodies to Mycobacterium tuberculosis**(MTB) HSP65 were present in 47% of SSc patients examined [10]. A SSc-RA overlap syndrome patient with Mycobacterium kansassi pulmonary infection showed improvement in both clinical symptoms and serum levels of IgM-RF and anti-mycobacterial HSP65 with anti-tuberculosis treatment [11]. Similarly, regression of skin changes in a patient with SSc following treatment for bacterial overgrowth with chronic ciprofloxacin has been reported [12]. The high homology between the microbial and human HSP65 proteins support a connection exists between tuberculosis and the development of SSc [10, 13]. There are Canadian, Russian and Norwegian cases describing temporal presentations of TB and SSc [13-15]. However, the majority of SSc patients do not have TB.

Various viruses, most prominently herpes and retroviruses, have been described with SSc [16-21]. One study found amino acid sequence homologies between both HIV-1 and HSV-1 proteins and autoimmune nuclear antigens, suggesting that co-expression of heterologous viruses having common immunosuppressive functions may generate auto-antibodies cross-reacting with certain nuclear proteins [16]. In 1979, there was a report of varicella occurring prior to diagnosis in a patient with SSc [18], although a later study found the frequency of varicella in SSc patients to be lower than that found in SLE patients (9.5% vs. 46.6%) [22]. Evidence of previous mononucleosis infection over a period of three years in 4 of 5 children suffering from localized SSc has been reported [17], and another study reported anti-fibrillarin antibodies in 58% of SSc patients and found that sequence homologies between fibrillarin, herpes virus type 1 and EBV proteins were suggestive of the notion that molecular mimicry may have played an important role in the induction of the antibodies [23]. Elevated levels of IgG antibodies to CMV and EBV titers have been found in patients with SSc [21]. Vaughan *et al* assayed SSc EBV and CMV in subjects with limited and diffuse SSc after observing that autoantibodies to the autoantigen p542 (presumed to be derived from the EBV antigen) occur in both mononucleosis and in SSc. Anti-CMV titers were significantly higher in the anti-p542 positive SSc patients than they were in anti-p542 negative patients. Anti-EBV titers were not different between those positive vs. negative for anti-p542, but were higher in all SSc groups than in a healthy control group, p<0.02. However, although CMV and EBV findings were not significantly different in skin biopsies from 13 SSc and 17 controls, investigators concluded that the incidence of the viruses was high, and they may be related to the process involved in the initiation of autoimmunity in SSc [24].

Borrelia burgdorferi (Bb) has been implicated in generalized morphea [25]. Additionally, an earlier study found 38% of seropositive (n=5) and 23% of seronegative (n=26) patients with morphea exhibited elevated B.burgdorferi induced lymphocyte proliferation, supporting a causal role, at least for some patients with morphea [26]. One study in Western Turkey (n=10 subjects with morphea and n=12 subjects with lichen sclerosus) found the presence of Bb DNA in 30% of morphea and 50% of lichen sclerosus patients suggesting a potential role in both conditions [27]; whereas, another study conducted in the U.S.A. (n = 35) did not find Bb spirochete in the pathogenesis of morphea and lichen sclerosus [28]. A more recent study reviewing the causal role of Bb in morphea concluded that previous conflicting results of European and American scientists could either be due to the fact that Bb is not a causative agent for morphea or that a subset of morphea is caused by a special subspecies of Bb present in Europe and Asia but not the U.S.A [29].

The evidence surrounding the potential importance of retroviruses in SSc is contradictory. One study of antibodies to retroviral proteins in SSc found that 8 of 29 SSc patients had antibodies to HIV proteins [20]. It was unclear whether a retrovirus was involved in the pathogenesis of SSc or whether the antibodies cross-reacted (molecular mimicry). Studies by Jimenez *et al* have demonstrated the ability of endogenous retroviruses to be expressed into proteins which can regulate cellular functions and lead to the occurrence of pathological conditions, but no conclusive evidence supporting pathogenesis for retroviruses in SSc was found [30, 31]. Other studies have found links with paramyxoviruses [32], congenital rubella [33], and parvovirus B19 [34].

In light of the existing body of research, it was expected that SSc patients would report more infections than a comparison group with non-inflammatory musculoskeletal (MSK) complaints prior to diagnosis, particularly in the 12 months prior to symptom onset. This study was designed to test if the self-reported frequency of certain infections and vaccinations was different between SSc and control groups, but also as an exploratory study to determine any potential infectious triggers (such as viruses or bacteria) that could be studied in greater depth in future research.

## MATERIALS AND METHODOLOGY

A five-page questionnaire consisting of 23 questions was developed to assess: whether subjects had ever had specific infections; whether they had had any infection or antibiotic exposure 12 months prior to their SSc diagnosis; travel history (and any associated illnesses) outside of North America in the 12 months preceding; vaccine history since 18 years of age; and whether or not they had ever been positive for a TB skin test. Specific questions addressed rubella, parvovirus, and herpes infections as well as rheumatic fever, illnesses that induced rash or fever, and previous hepatitis A, B and C exposure. The questionnaire was approved by the University of Western Ontario Health Sciences Research Ethics Board and it was pre-tested on clinic patients (controls) to ensure that it was understandable and that no consistent misinterpretations were being made. The questionnaire had been validated previously in a study of systemic lupus erythematosus (SLE) patients in the same rheumatology practice, which reported an increased frequency of herpes zoster clustered around the time of diagnosis with SLE [35].

The rheumatologist who conducted this research, based in London, Ontario, is a clinical expert in scleroderma servicing Southwestern Ontario. Referrals are from a large population base of approximately one million, but also from more geographically remote northern areas. Although many of the SSc cases from the referral catchment area are seen in the London clinic, not all cases will be recognized and referred to tertiary care, and other cases may be followed elsewhere. The challenge of obtaining a representative study sample of SSc from clinical cohorts has been described in the literature and although estimates vary, it is generally accepted that less severe cases will generally be under-represented [36-38].

Patient selection was completed using the computerized billing system to identify all patients who had been seen in the clinic for SSc over the past 2 years and who were current patients with accurate addresses. Controls with non-inflammatory musculoskeletal disorders were subsequently chosen at a ratio of 4 controls:1 SSc patient using the same method (a billing list based on date of last clinic visit who had been seen for non-inflammatory MSK conditions). Controls were over-sampled as it was expected that the response rate would be less. The questionnaire was sent to scleroderma subjects (n=83, cases) and control subjects (n=351), who had non-inflammatory MSK disorders such as osteoarthritis (n=204), tendonitis (n=58) and fibromyalgia (n=89), from the same practice.

All subjects were blinded as to the hypothesis and were sent questionnaire packages that included a stamped return addressed envelope, a letter of information and the questionnaire. Written informed consent was obtained from the study participants. Data were entered and statistical analyses were performed using SAS-based JMP statistical software (Version 4.0) [39]. Trends in survey responses were studied. For each survey question, responses of uncertainty were excluded from the analyses. Group means, odds ratios and 95% confidence intervals were calculated for exposures of interest.

In order to further characterize the SSc cohort of subjects a post hoc chart review was conducted to obtain information on type of SSc (limited vs. diffuse), disease duration, age at diagnosis with SSc, and the Modified Rodnan skin score (MRSS) (MRSS ranges from 0 to 78 with lower scores representing less skin involvement) [40] and Health Assessment Questionnaire Disability Index (HAQ-DI) (HAQ-DI ranges from 0-3 with higher scores indicating more functional disability) [41].

## RESULTS

The response rate in the SSc group was 78% (n=65) and in the controls it was 56% (n=196).As can be seen in Table **1**, the mean age was 56 ± 1.6 in the SSc group and 58 ± 0.9 in the controls (MSK); 88% and 82% were female in the SSc and MSK groups. The SSc group was comprised of 37% diffuse SSc, had a mean overall MRSS score of 9.5 ± 1.63, and a mean HAQ-DI score of 1.12 ±0.106 (Table **2**). The mean disease duration for the SSc group was 9.75 ± 0.53 years, and the mean age of diagnosis was 50.05 ± 1.64 years old (Table **2**). Patients were primarily Caucasian; a very few were Native Canadian, Asian or African Canadians.

MRSS and HAQ-DI values were determined by subsequent chart review to further characterize the cohort of SSc subjects who responded to this research questionnaire. Scores were available for only 28 and 45, respectively, of the 65 subjects who participated in the research study.

The data in Table **3** show that no association between recalled symptomatic prior infections and scleroderma was observed. For several types of infections numbers were small and results were not statistically significant, and thus, there were no meaningful differences in reported infections between the cases and controls. Frequencies in controls compared to SSc cases included: hepatitis A (3% vs. 1.6%, p=0.6), hepatitis B (0% vs. 1.6%, p=0.08), hepatitis C (2% vs. 1.6%, p=0.8), hepatitis of any kind (including any of A, B, C or if unable to recall which type; 7% vs. 5%, p=0.4), rubella (58% vs. 51%, p=0.4), any bacterial infection (78% vs. 68%, p=0.1), positive tuberculosis skin test (13% vs. 8%, p=0.2), past rheumatic fever (7% vs. 8%, p=0.8), and herpes zoster prior to disease diagnosis (8% vs. 12%, p=0.4). Subjects with non-inflammatory MSK disorders were significantly more likely to report a previous joint trauma (44% vs. 19%, p=0.0002), and this finding was used as internal validation where those with non-inflammatory MSK conditions should have more preceding trauma.

Data focusing on the 12-month period prior to diagnosis of SSc or non-inflammatory MSK disorders demonstrated that controls were significantly more likely than SSc subjects to report having experienced any infection within the 12 months prior to disease diagnosis (35% vs. 16%, p<0.006). Controls reported that within the year prior to disease diagnosis, they had an increased occurrence of: streptococcal infections (13% vs. 8%, p<0.3), infections with diarrhea and vomiting (15% vs. 10%, p<0.3), and antibiotic use (50% vs. 34%, p<0.09), although none of these questions were statistically significant (Table **3**).

## DISCUSSION

We found no trends of increased self-reported infection in SSc prior to symptom onset compared to controls. In contrast to the literature, our findings did not support that more subjects with SSc had been exposed to TB, but the prevalence of TB exposure was very low in our series. We did not have Lyme disease in either the cases or the controls, as it is rare in Southwestern Ontario. Since there were no signals of increased infections we did not subset our patients into diffuse and limited, as exploratory analyses were not warranted. However, patients with limited SSc may have a long time between first symptoms and diagnosis.

Consistent with the concept that herpes family viruses may be involved in the development of autoimmunity without being the specific triggering event, we have also reported a temporal clustering of varicella zoster virus (VZV) reactivation around the time of disease diagnosis in patients with systemic lupus erythematosus, whereby VZV infections were clustered just prior to or after diagnosis in SLE but were more widely temporally spaced in the controls (the mean time until VZV reactivation in SLE subjects was 1 ± 4.5 years after the diagnosis of SLE, compared to controls which was 14.7 ± 4 years before the diagnosis of non-inflammatory MSK disorder; p<0.003) [35]. However, no longitudinal study has demonstrated that a viral illness can increase the risk of scleroderma (2). Fifty SSc patients were negative for anti-HEV antibodies (all patients tested), suggesting that hepatitis E is not involved in the pathogenesis of SSc [42].

This case control survey study provided no evidence that the occurrence of infectious events (either bacterial or viral) was more common in the one-year period prior to the development of SSc compared to controls with non-inflammatory MSK conditions. In fact, the study controls were more likely to report having had a bacterial infection ever prior to diagnosis, or any infection in the one-year prior to disease diagnosis. Although it appears that there is no relationship between self-reported infectious exposures and the subsequent development of SSc, this relationship may be modified by genetics or other unknown factors. If this paradigm holds, then only certain susceptible people would develop SSc following exposure to an infectious agent. Thus, the rates of infections could be similar between the cases and controls, but the underlying genetics (or other unknown modifying factors) could alter the response to the infectious agent in a way that would result in the development of SSc only in those with a certain genetic complement. It is also possible that genetic factors, as well as several types of exogenous factors, could be required for scleroderma to develop, and hence infectious triggers may be involved in the etiology of SSc but exert an effect as part of a complicated process of disease development. 

Our data do not support previous studies that found a causal link between infectious triggers and the subsequent development of SSc; but SSc patients were slightly (but not significantly) more likely than controls to report previous herpes zoster infections, and others have also observed this [2]. A case control study using objective evidence of past infection with herpes zoster will be needed to clarify the link between the two. Given that IgG antibodies have been found at increased levels in certain herpes viruses in scleroderma [2], and herpes viruses have been detected in SSc patients in several case studies [18, 22], and that studies have found sequence homologies between herpes virus and endogenous proteins [21], the possible association between herpes viruses and SSc merits further research.

This study has limitations. These include the lower response rate in the control group, and that recall bias may have occurred. The differential response rate in the controls is likely due to the fact that the SSc patients in this cohort were followed regularly (usually twice a year), whereas many OA or other non-inflammatory patients are seen less often (if followed at all) and may be less apt to complete a questionnaire. Additionally, those with SSc (especially diffuse SSc) may attach more importance to research to identify the etiology of their potentially life-threatening disease compared to patients with OA or other MSK conditions who may have been told that their disease has at least partially been caused by trauma or “wear and tear” of joints.

Further limitations included that this retrospective study had patients remember infections 1-year prior to diagnosis, which may have occurred (on average) more than nine years ago. However, patients often search for answers and may over interpret events that occurred prior to disease onset, so the bias should have been that more infections were found in the SSc group due to a diagnosis of a serious disease. Patients may have misinterpreted survey questions, but this should not have been different between the two groups. If there was recall bias, we would have expected it to occur in favour of our hypothesis of increased infection in SSc; however, this was not the case. Additionally, asymptomatic infections would not be reported in either group. Diagnostic and viral serologic tests were not done. However, past research has been conducted for which self-report of medical conditions by subjects with SSc has been validated [43].

## CONCLUSION

Self-report of infections prior to diagnosis in SSc is not increased compared to non-inflammatory controls. This study does not support that self-report of symptomatic infections are more likely to occur prior to diagnosis ever or within 1-year prior to symptom onset of SSc, or that vaccinations in adulthood trigger SSc.

## Figures and Tables

**Table 1:** Demographic Factors for Subjects with SSc or Non-Inflammatory MSK Disorders. Given as Percentage (N = Total Num-ber of Subjects who Responded, with Missing Data Excluded)

	Scleroderma % (N)	Controls % (N)	p-value
Respondents	78 (65)	56 (196)	0.0002
*Age (mean ±SEM)*	56 ± 1.6	58 ± 0.9	0.2
Female gender	88 (57)	82 (160)	0.2

**Table 2.** Characteristics of the Subjects in the London, Ontario SSc Cohort who Responded to the Study Questionnaire. Given as Mean ± SEM or as Percentage (Where Applicable). The Total Number of Subjects for whom a Measurement of the Characteristic was Available has been Indicated in Parentheses.

Characteristics of the SSc Study Sample (N=65)	N	Proportion/Mean	Min, Max
Diffuse disease	65	37% (65)	-
Disease duration	64	9.75 ± 0.53	2, 26
Age at diagnosis	64	50.05 ± 1.64	21, 80
Modified Rodnan Skin Score (MRSS) * *	28	9.5 ± 1.63	2, 41
*Health Assessment Questionnaire Disability Score (HAQ-DI)*	45	1.12 ±0.106	0, 2.625

**Table 3.** Infections, Vaccinations and/or Trauma Experienced (Ever) Prior to Diagnosis, or One-Year Prior to Diagnosis of SSc/Non-Inflammatory MSK Disorder. Given as Percentage (N).

Infection(s) Ever Prior to Diagnosis	Scleroderma % (N)	Controls % (N)	p-value	OR Scl vs. controls	95 % CI
Bacterial infections	68 (59)	78 (177)	0.1	0.59	(0.31,1.14)
Hepatitis A	1.6 (62)	3 (186)	0.6	0.59	(0.07,5.18)
Hepatitis B	1.6 (62)	0 (186)	0.08	-	-
Hepatitis C	1.6 (62)	2 (186)	0.8	0.74	(0.08,6.80)
Herpes zoster	12 (60)	8 (192)	0.4	1.45	(0.57,3.72)
Parvovirus	1.6 (62)	0 (186)	0.2	-	-
Rheumatic fever	8 (62)	7 (186)	0.8	1.17	(0.40,3.40)
Rubella	51 (49)	58 (144)	0.5	0.76	(0.40,1.47)
Tuberculosis skin test	8 (64)	13 (188)	0.2	0.55	(0.20,1.51)
Vaccinations	49 (59)	50 (189)	0.9	0.95	(0.53,1.72)
Trauma prior	19 (64)	44 (195)	0.0003	0.30	(0.15,1.59)
**Infections 1-Year Prior to Diagnosis**	**Scleroderma % (N)**	**Controls % (N)**	**p-value **	**OR Scl vs. controls**	**95% CI**
Any infection	16 (51)	35 (163)	0.006	0.34	(0.15,0.78)
Streptococcal throat infection	8 (53)	13 (157)	0.3	0.53	(0.17,1.62)
Antibiotic use	34 (35)	50 (111)	0.09	0.51	(0.23,1.13)

Please note: the total number of subjects (N) who responded to each question was variable, as subjects may have skipped questions for which they were uncertain, and if they indicated that they were unsure, they were excluded from the analysis for that item.
